# Toxicological Effects of Thimerosal and Aluminum in the Liver, Kidney, and Brain of Zebrafish (*Danio rerio*)

**DOI:** 10.3390/metabo13090975

**Published:** 2023-08-27

**Authors:** Maria Eduarda Andrade Galiciolli, Juliana Ferreira Silva, Maritana Mela Prodocimo, Henrique Aparecido Laureano, Sabrina Loise de Morais Calado, Claudia Sirlene Oliveira, Izonete Cristina Guiloski

**Affiliations:** 1 Instituto de Pesquisa Pelé Pequeno Príncipe, Avenida Silva Jardim, 1632, Água Verde, Curitiba 80250-200, PR, Brazil; maria.galiciolli@aluno.fpp.edu.br (M.E.A.G.); juliana.silva@aluno.fpp.edu.br (J.F.S.);; 2 Faculdades Pequeno Príncipe, Curitiba 80230-020, PR, Brazil; 3 Departamento de Biologia Celular e Molecular, Universidade Federal do Paraná, Centro Politécnico, Avenida Cel. Francisco H. dos Santos, 100—Jardim das Américas, Curitiba—PR, Curitiba 81531-980, PR, Brazil; maritana.mela@gmail.com; 4 Aplysia Assessoria e Consultoria Ltd., Rua Júlia Lacourt Penna, 335, Jd. Camburi, Vitória 29090-210, ES, Brazil

**Keywords:** metals, vaccine, preservative, adjuvants, biomarkers, histopathology

## Abstract

Vaccination programs in the first years of a child’s life are effective and extremely important strategies for the successful eradication of diseases. However, as no intervention is without risks, the metal-based components of some vaccines, such as thimerosal (TMS), a preservative composed of ethylmercury, and aluminum (Al), have begun to generate distrust on the part of the population. Therefore, this study evaluated the effects of exposure to thimerosal and aluminum hydroxide (alone or in mixture) on *Danio rerio* (zebrafish) specimens. The fish were exposed to thimerosal and/or aluminum hydroxide intraperitoneally. The liver, kidney, and brain were removed for a biochemical biomarker analysis, histopathological analysis, and metal quantification. As a result, we observed changes in the activity of the analyzed enzymes (SOD, GST, GPx) in the kidney and brain of the zebrafish, a reduction in GSH levels in all analyzed tissues, and a reduction in MT levels in the kidney and liver as well as in the brain. Changes in AChE enzyme activity were observed. The biochemical results corroborate the changes observed in the lesion index and histomorphology sections. We emphasize the importance of joint research on these compounds to increase the population’s safety against their possible toxic effects.

## 1. Introduction

Vaccination is a relevant tool in terms of infectious disease control and eradication. Vaccines are essential, especially in developing countries, where sanitary conditions and public health need to be improved [1,2]. However, with the decrease in vaccine-preventable diseases, the risks of adverse effects caused by vaccines, especially regarding the substances used in their composition, have been explored further [3,4,5,6].

Besides the antigen, vaccines may have in their composition adjuvants (e.g., aluminum hydroxide) and preservatives (e.g., thimerosal). Adjuvants are employed to increase the effectiveness of the individual’s immune response, allowing for a lower antigen dose [7,8]. Adjuvants activate innate immune cells; however, the action mechanism has not been fully specified yet [7,9,10]. Conversely, preservatives are added to the formulation of multidose vaccines to prevent microorganism contamination [8,11,12,13]. Although these compounds reduce the pharmaceutical industry’s costs of packaging, transport, and storage and use a lower antigen dose [1], the administration of these compounds can cause adverse effects, such as allergic and inflammatory reactions and possible systemic effects [14,15].

Aluminum hydroxide has a high potential as an immunological adjuvant and is used in the formulation of some vaccines, such as hepatitis B, triple bacterial, and meningococcal B [16]; however, aluminum can induce side effects in the body. These effects include the triggering of an autoimmune response due to the high capacity of the metal to bind to proteins, causing modifications in autogenous epitopes, leading to the recognition of these proteins as foreign by T cells and the consequent production of autoantibodies and hypersensitivity reactions at the vaccine application site [7,17,18].

Thimerosal contains a mercury (Hg) atom bound to a thiosalicylate moiety and an ethyl group [19]. Thimerosal became part of the composition of cosmetics, vaccines, and pharmaceutical products, such as Merthiolate^®^, due to its fungicidal and antibacterial properties [20,21]; however, some researchers have suggested that thimerosal is more toxic to human cells than to bacteria [22]. Inside the organism, the mercury–sulfur bond of the thimerosal molecule breaks, releasing thiosalicylate and ethylmercury (EtHg) [23]. Mercury is a widely recognized neurotoxic chemical element, mainly in its organic chemical forms (e.g., methylmercury and EtHg) [13,24,25]. Developing children are especially vulnerable to neurotoxic agents, so exposure to Hg-containing molecules needs to be limited or null [13]. Thus, studies exploring the impact of thimerosal and consequently EtHg exposure are pivotal [5,26].

The *Danio rerio* is a species of tropical fish that has been used as an alternative animal model for research due to its genetic similarity with human genes (70%) [27]. Because of this, several studies involving numerous toxic agents are being developed to define genetic and toxicity mechanisms and neurobehavioral effects using this experimental model [28,29]

Since several studies have explored the toxicity of aluminum hydroxide [30,31,32,33] and isolated thimerosal [21,34,35,36,37] and these compounds are found together in the formulation of some multidose vaccines applied in developing countries, such as Brazil [4], it is relevant to study their toxicological effects in combination. Thus, this study aimed to analyze the toxic effects of metal-containing molecules used as adjuvants and preservatives (aluminum hydroxide and thimerosal, respectively) in the formulation of vaccines using *D. rerio* as an experimental model.

## 2. Materials and Methods

### 2.1. Animals

Adult (~90 days) wild-type zebrafish (*Danio rerio*; both sexes) (375 fish; 0.241 ± 0.072 g; 2.9 ± 0.29 cm) were obtained from a commercial supplier (Planeta Aquários; Curitiba, PR, Brazil) and acclimated for at least two weeks in thermostatic glass aquariums (2 L, with a maximum of 5 fish per liter) before experiments. During the exposure period, fish remained in 2 L glass aquariums (*n* = 5 per aquarium) with constant aeration, a photoperiod of 14:10 h, and an approximate temperature of 26 °C. Animals were fed *ad libitum* once a day with commercial floccular food (Sera Vipagran^®^). The excrement at the bottom of the aquarium was removed every 48 h by siphoning. All animals used were free of any signs of disease and maintained in accordance with the Brazilian Institute of Health Guide for Care and Use of Laboratory Animals. This research was approved by the Ethical Committee on the Use of Animals of Instituto de Pesquisa Pelé Pequeno Principe, Curitiba-PR, under protocol number 058-2020.

### 2.2. Experimental Conditions

The water in the aquariums was monitored from the first to the last day of the experiments to verify the parameters of pH, ammonia, nitrite, chlorine, hardness (using Labcon^®^ commercial kits), and temperature.

### 2.3. Experimental Design

#### 2.3.1. Thimerosal and Aluminum Hydroxide

To prepare the solutions, 7.5 mg of thimerosal (Sigma-Aldrich^®^, St. Louis, MO, USA; MW: 404.81 g/mol) (TMS) and 175 mg of aluminum hydroxide (Sigma-Aldrich^®^, St. Louis, MO, USA; MW: 78.00 g/mol) (Al) were weighed and dissolved in 50 mL of saline (NaCl, 0.9%; Farmace^®^, Barbalha, CE, Brazil) (Sal; vehicle). The doses were prepared from the stock solutions.

#### 2.3.2. Mortality Test

To verify the possible mortality induced by TMS or Al, 55 fish were divided into 11 groups (*n* = 5/group) (Table S1). Doses were based on the sum of a known quantity of TMS and Al present in mandatory multidose vaccines for children from 0 to 10 years old in Brazil, namely, 0.30 mg TMS/child and 7.0 mg Al/child (ANVISA 2013) (Table S2). From this, the dose curve was produced (0, 0.012, 0.06, 0.3, 1.5, and 7.5 mg TMS/kg of fish and 0, 0.28, 1.40, 7.0, 35.0, and 175.0 mg Al/kg of fish). Each fish was weighed before injection to adjust the dosage. Animals were anesthetized with 0.001% benzocaine (Sigma-Aldrich^®^, St. Louis, MO, USA) and exposed to TMS and/or Al through a single intraperitoneal injection using an insulin syringe with a needle size of 13 mm × 0.45 mm (Descarpack^®^, São Paulo, SP, Brazil). The injected volume did not exceed 10 µL per fish. The mortality was checked every 24 h for 96 h. As no mortality was observed (Table S3), only 7.5 mg TMS/kg of fish and 175 mg Al/kg of fish were evaluated in the following tests.

#### 2.3.3. Exposure to TMS and/or Al

**Figure 1:**
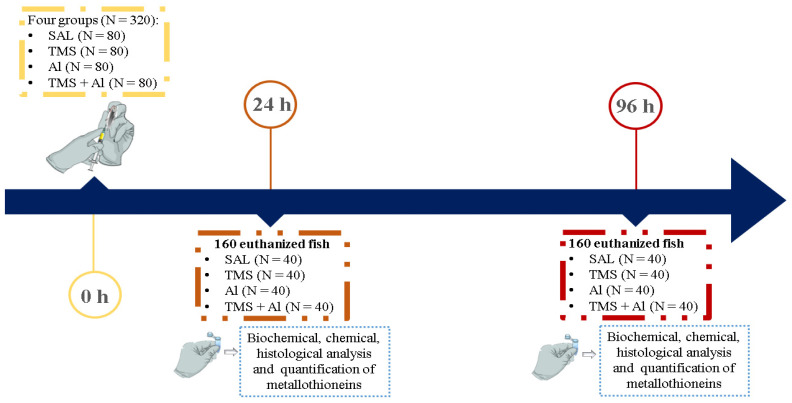
Experimental design. SAL: 0.9% saline; TMS: thimerosal (7.5 mg/kg); Al: aluminum hydroxide (175.0 mg/kg).

Three hundred and twenty fish (*n* = 80 per group) were randomly divided into the groups: control (Saline 0.9%; Sal), thimerosal (7.5 mg/kg; TMS), aluminum (175.0 mg/kg; Al), and thimerosal + aluminum (TMS + Al). Each fish was weighed before injection to adjust the dosage. Animals were anesthetized with 0.001% benzocaine and exposed to TMS and/or Al through a single intraperitoneal injection using an insulin syringe with a needle size of 13 mm × 0.45 mm. Twenty-four and ninety-six hours after the exposure, 40 specimens per group were anesthetized with 0.001% benzocaine, weighed, measured, and euthanized by vertebral section for tissue collection (liver, kidney, muscle, and brain). The tissues were properly separated for histological (*n* = 10/group), biochemical (*n* = 10/group), chemical (*n* = 5/group) analyses as well as the quantification of metallothionein (*n* = 15/group) (Figure 1).

### 2.4. Analysis of Biochemical Biomarkers

#### 2.4.1. Sample Preparation

The liver, kidney, and brain were collected under ice, homogenized in 100 mM sodium phosphate buffer with a pH 7.0 and centrifuged at 4 °C (12,000 rpm—15 min). The supernatants were aliquoted for analysis of total protein, superoxide dismutase (SOD), glutathione S-transferase (GST), glutathione peroxidase (GPx), and reduced glutathione (GSH). An aliquot with the brain homogenate was also performed for the analysis of acetylcholinesterase (AChE) activity. Aliquots were kept at −80 °C until the analysis.

#### 2.4.2. Quantification of Total Protein

Proteins were quantified using the method of Bradford [38]. A dilution (1:2; *v/v*) was performed for each tissue. Subsequently, 10 μL of each sample was added in triplicate in a 96-well plate, and 250 μL of Bradford reagent (Sigma-Aldrich^®^, St. Louis, MO, USA) was added to each well at room temperature. Bovine serum albumin (Sigma-Aldrich^®^, St. Louis, MO, USA) was used to prepare the standard curve (0–1000 μg/mL). The reading was performed at a wavelength of 595 nm.

#### 2.4.3. Superoxide Dismutase (SOD) Activity

For the analysis of SOD activity, the method proposed by Gao et al. [39] was used, which is based on the ability of SOD to inhibit pyrogallol auto-oxidation. The buffer (885 μL, 1 M Tris, 5 mM EDTA, pH 8.0) was added in an aliquot of the sample (40 μL), followed by vortexing; after this process, 50 μL of 15 mM pyrogallol (Neon^®^, Suzano, SP, Brazil) was added and incubated for 30 min at room temperature. After incubation, 25 μL of 1 N hydrochloric acid (CRQ^®^, Casa Grande, SP, Brazil) was added to stop the reaction. The samples (200 μL) were pipetted into a 96-well plate, and the absorbance was measured at 440 nm; the results were expressed in U of SOD/mg of protein.

#### 2.4.4. Glutathione S-Transferase (GST) Activity

We followed the technique described by Keen et al. [40] with modifications. Briefly, samples (20 μL) were pipetted in triplicate into 96-well microplates, followed by 180 μL of a solution containing 3.0 mM 1-chloro-2,4-dinitrobenzene—CDNB (Sigma-Aldrich^®^, St. Louis, MO, USA), 3.0 mM reduced glutathione—GSH (Sigma-Aldrich^®^, St. Louis, MO, USA), and 0.1 M phosphate buffer (pH 6.5) at room temperature. Absorbance was measured at 340 nm for 5 min, with one-minute intervals between each reading. Enzyme activity was expressed in nmol/min/mg of protein.

#### 2.4.5. Glutathione Peroxidase (GPx) Activity

The methodology was based on the technique described by Paglia and Valentine [41]. Briefly, 10 μL of sample and 130 μL of solution 1 (0.1 M sodium phosphate buffer, pH 7.0, 3.07 mM sodium azide (Labsynth^®^, Diadema, SP, Brazil), 0.30 mM NADPH (Sigma-Aldrich^®^, St. Louis, MO, USA), 3.07 mM GSH, and 1.54 U/mL glutathione reductase (Sigma-Aldrich^®^, St. Louis, MO, USA) were added to microtubes and incubated for 2 min at room temperature. Afterwards, 60 μL of solution 2 was added (5 mM hydrogen peroxide (Neon^®^, Suzano, SP, Brazil) in 0.1 M sodium phosphate buffer, pH 7.0). The absorbance reading was performed at 340 nm for 5 min, with one-minute intervals between each reading. Enzyme activity was expressed in nmol/min/mg of protein.

#### 2.4.6. Acetylcholinesterase (AChE) Activity

The AChE activity was measured based on the methodology described by Ellman et al. [42]. Sample (25 μL) was mixed with 0.75 mM 5,5-dithio-bis-2-nitrobenzoic acid (DTNB; Sigma-Aldrich^®^, St. Louis, MO, USA) prepared in 0.1 M potassium phosphate buffer (pH 7.5) and 10 mM acetylthiocholine iodide (Sigma-Aldrich^®^, St. Louis, MO, USA) at room temperature. The absorbance reading was performed at 405 nm for 5 min, with one-minute intervals between each reading. Results were expressed as nmol/min/mg of protein.

#### 2.4.7. Reduced Glutathione (GSH) Concentration

The determination of GSH followed the method described by Sedlak and Lindsay [43]. The samples’ proteins were precipitated with 50% trichloroacetic acid (Neon^®^, Suzano, SP, Brazil), and the supernatants were used for analysis. Into microplates, 50 μL of samples, 230 μL of 0.4 M Tris-base buffer (Labsynth^®^, Diadema, SP, Brazil) with a pH 8.9, and 20 μL of 2.5 mM DTNB dissolved in methanol were added (Química Moderna^®^, Barueri, SP, Brazil). A GSH standard curve (0–80 μg/mL) was used for comparison. The absorbance reading was performed at 415 nm. The results are presented as μg/mg of protein.

#### 2.4.8. Quantification of Metallothionein (MT)

For MT assays, a pool of 5 samples of the liver and kidney were homogenized in 20 mM Tris-HCl (J.T. Baker^®^, Radnor, PA, USA) buffer at a pH 8.6, containing 0.5 mM phenylmethylsulfonyl fluoride (Sigma-Aldrich^®^, St. Louis, MO, USA) as antiproteolytic agent and 0.01% β-mercaptoethanol (Sigma-Aldrich^®^, St. Louis, MO, USA) as a reducing agent. After being centrifuged at 15.000 g for 30 min at 4 °C, the supernatant was used for the determination of MT, according to Viarengo et al. [44], using the colorimetric method with Ellman’s reagent. The reading was performed with a spectrophotometer at 412 nm, and the result was presented as µg/mg of protein.

### 2.5. Histopathological Analysis

The samples destined for histopathological analysis were chemically preserved in ALFAC (80% ethanol, 15% formalin, and 5% acetic acid) for 16 h and kept in 70% ethanol at 4 °C until the preparation of the slides. The samples underwent dehydration (10 h), clarification (2 h), and infiltration by paraffin at 65 °C (2 h) in an automated device (OMA Tissue Processor—Model D40; São Paulo, SP, Brazil). For the cut, a high-profile disposable blade (Leica brand) was used at an angle of 45°, with manual rotary cutting speed and dry-sectioned, sequential cuts with a thickness of 3 µm (equipment: American Optical Manual Rotary Microtome—Model: 820—Series: 54637, Buffalo, NY, USA). Subsequently, the samples were extended in a histological bath containing water at 50 °C. The cuts were adhered to glass slides and placed in an oven at 65 °C for 30 min. The slides were submerged in xylene at 65 °C to remove the paraffin for 5 min. Slides were stained with Harris Hematoxylin (3 min), running water (5 min), 2% eosin Phloxin (2 min), and running water again (to remove excess dye), followed by the dehydration and clarification process and finishing by sealing them with Canada Balsam and a coverslip. In the slides, the nuclei are in blue, the cytoplasm is in pink, and other structures are in pink.

The injury index based on histopathological findings was calculated according to Bernet et al. [45] and modified by Mela et al. [46]. Briefly, the lesions and alterations in tissue are classified in categories according to their biological importance (1—minimal, easily reversible; 2—moderate, reversible in most cases; and 3—marked, generally irreversible) and severity, with scores from 0 to 6. The injury index for each group of lesions in tissues was calculated using the formula: *Iorg. = ∑rp ∑alt (a* × *w)*, where *org* represents the organ (constant), *rp* the reaction pattern, *alt* the alteration, *a* the score value, and *w* the importance factor of the lesion. The analysis was blindly conducted.

### 2.6. Metal Quantification

Total Hg and Al were determined in *Danio rerio*’s head and body by atomic absorption spectrometry (AAS) with atomization in a graphite oven (GF-AAS) and cold steam (CV-AAS) using Analytik Jena spectrometers (NovAA 300 models with hydride generator module HS60 and ContrAA 700). The analyzed samples underwent pre-treatment for total digestion in a microwave oven (Anton Paar brand, Graz, Austria). The samples were digested by acid using the oxidant mixture HNO_3_ + H_2_O_2_ according to the EPA3050B reference method described by the EPA [47]. After total digestion, the samples were diluted to 25 mL in ultrapure water (Milli-Q) and analyzed according to the methodologies validated for each element. For the determination of total Hg, mol/L HCl and NaBH_4_ (0.25% *w/v*) solutions were used as acidic and reducing media, respectively. Hollow cathode lamps were operated at 4 mA. The wavelength was set to 253.7 nm for Hg and 309.3 nm for Al, and the spectral band passes at 0.5 nm for both metals. The final concentration of each element was calculated in µg/g according to the wet mass used in each digested sample (~300 mg).

### 2.7. Statistical Analysis

The data were statistically analyzed using Prism GraphPad software, version 6.0, using the Kruskal–Wallis test followed by Dunn’s test and presented as median ± interquartile interval (histopathological analysis) or using one-way ANOVA followed by Dunnet’s test and presented as mean ± SEM (biochemical and chemical analyses and quantification of metallothioneins). The choice of the parametric or non-parametric test was defined after carrying out the Shapiro normality distribution test. Results were considered statistically different when *p* < 0.05.

The Principal Component Analysis was performed with the R (R Code Team 2022) language and environment for statistical computing. The following R packages were used: {dplyr} [48], {ggplot2} [49], {ggfortify} [50,51], and {patchwork} [52].

## 3. Results

### 3.1. Experimental Conditions

There was no mortality during the experiment. The water parameters were as follows: a pH 7 ± 0.6, 0.04 ppm ammonia, 0 ppm nitrite, a hardness of 60 mg/L, and a temperature of 25 ± 2 °C. All the water parameters were determined in triplicate, and the values did not differ among the groups.

### 3.2. Biochemical Analysis

**Figure 2:**
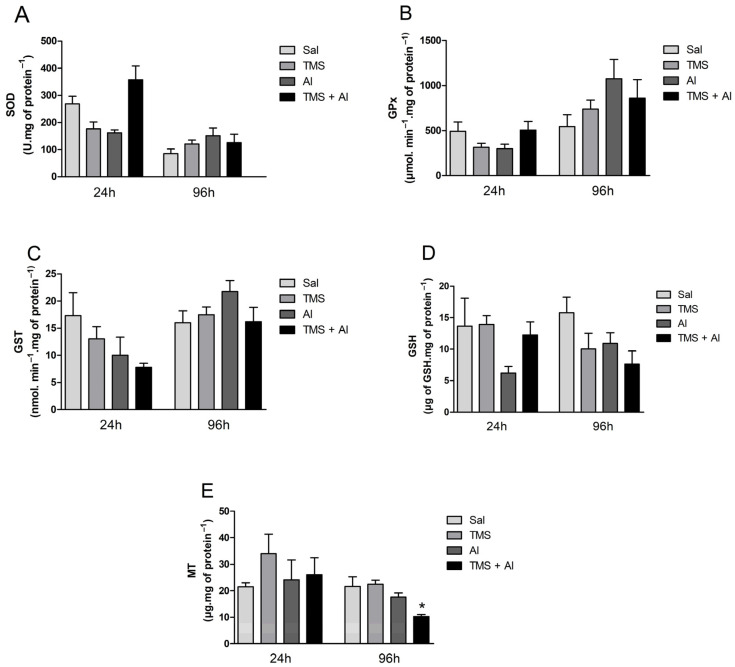
Hepatic SOD (**A**), GPx (**B**), and GST (**C**) activity and GSH (**D**) and MT (**E**) levels in *D. rerio* exposed intraperitoneally to Sal (saline, 0.9%), TMS (thimerosal, 7.5 mg/kg), Al (aluminum hydroxide, 175.0 mg/kg), and TMS + Al (7.5 mg TMS/kg + 175.0 mg Al/kg) 24 h and 96 h after exposure. Results were analyzed by one-way ANOVA followed by Dunnett’s post-hoc test and are presented as mean ± standard error (biochemical biomarkers *n* = 6–10; metallothionein *n* = 3). * = statistically significant difference from the control group (Sal).

The SOD, GPx, and GST hepatic activity are shown in Figure 2A–C. The one-way ANOVA revealed the TMS and/or Al exposure had no effect on the hepatic enzyme activities 24 h after exposure. The hepatic GSH and MT contents are shown in Figure 2D,E. The one-way ANOVA revealed the metals had no effect on the GSH and MT hepatic levels 24 h after exposure. Ninety-six hours after exposure, the TMS and/or Al exposure had no effect on the hepatic enzyme activities. On the other hand, the one-way ANOVA revealed an effect of the metals on MT hepatic contents 96 h after exposure (F (3.5) = 10.21; *p* = 0.0143). The exposure to TMS + Al caused a significant decrease in the hepatic MT content when compared to that of non-exposed fish.

**Figure 3:**
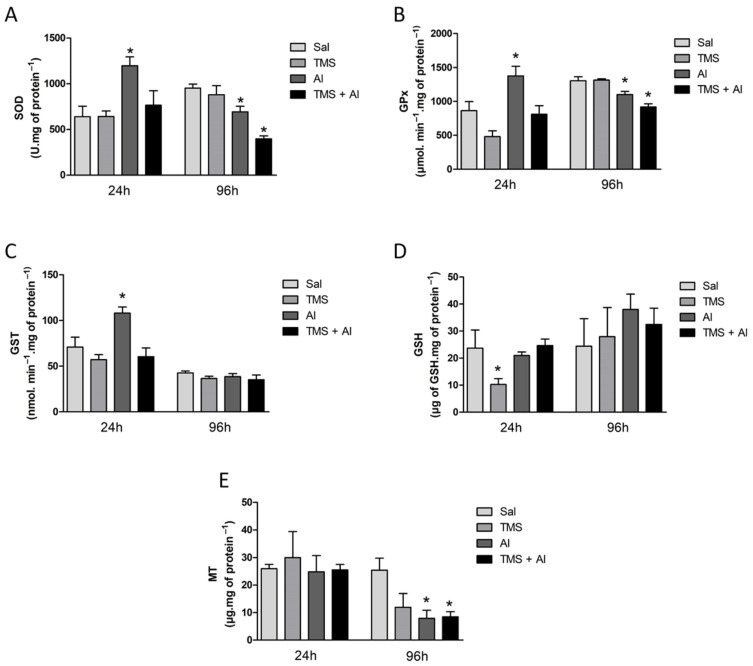
Kidney SOD (**A**), GPx (**B**), and GST (**C**) activity and GSH (**D**) and MT (**E**) levels in *D. rerio* exposed intraperitoneally to Sal (saline, 0.9%), TMS (thimerosal, 7.5 mg/kg), Al (aluminum hydroxide, 175.0 mg/kg), and TMS + Al (7.5 mg TMS/kg + 175.0 mg Al/kg) 24 h and 96 h after exposure. Results were analyzed by one-way ANOVA followed by Dunnett’s post-hoc test and are presented as mean ± standard error (biochemical biomarker *n* = 6–10; metallothionein *n* = 3). * = statistically significant difference from the control group (Sal).

The SOD, GPx, and GST renal activities are shown in Figure 3A–C. The one-way ANOVA revealed a significant increase in renal SOD (F (3.22) = 5.794; *p* = 0.0045), GPx (F (3.22) = 9.947; *p* = 0.0002), and GST (F (3.22) = 6.336; *p* = 0.0029) activities in the Al group 24 h after exposure. The renal GSH and MT content is shown in Figure 3D,E. The one-way ANOVA revealed a significant decrease in GSH levels (F (3.22) = 4.954; *p* = 0.0089) in the fish exposed to TMS (the TMS group) 24 h after exposure. Ninety-six hours after exposure, a significant decrease in the SOD (F (3.35) = 14.15; *p* < 0.0001) and GPx (F (3.34) = 15.80; *p* < 0.0001) activities and in the MT content (F (3.8) = 4.724; *p* = 0.0351) was observed in the animals exposed to Al (the Al and TMS + Al groups).

**Figure 4:**
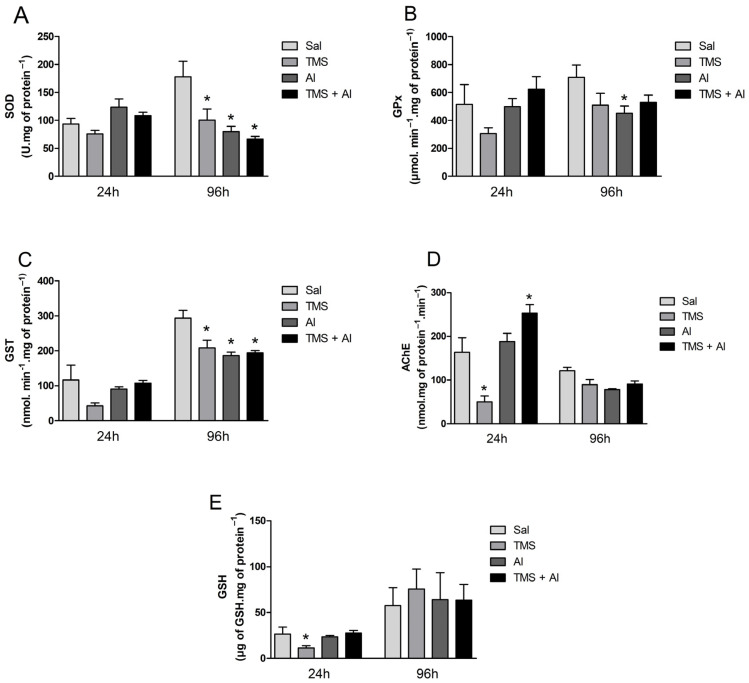
Brain SOD (**A**), GPx (**B**), GST (**C**), and AChE (**D**) activity and GSH (**E**) levels in *D. rerio* exposed intraperitoneally to Sal (saline, 0.9%), TMS (thimerosal, 7.5 mg/kg), Al (aluminum hydroxide, 175.0 mg/kg), and TMS + Al (7.5 mg TMS/kg + 175.0 mg Al/kg) 24 h and 96 h after exposure. Results were analyzed by one-way ANOVA followed by Dunnett’s post-hoc test and are presented as mean ± standard error (*n* = 6–10) * = statistically significant difference from the control group (Sal).

The biochemical markers (the SOD, GPx, GST, and AChE activities and GSH content) measured in the brain are shown in Figure 4A–E. The one-way ANOVA revealed a significant decrease in the GSH levels 24 h after exposure to TMS only (F (3.22) = 4.953; *p* = 0.0089). The one-way ANOVA revealed an effect of the TMS exposure on the AChE activity 24 h after exposure (F (3.23) = 20.15; *p* < 0.0001). The fish exposed only to the TMS (the TMS group) presented a statistically significant decrease in AChE activity. Interestingly, the enzyme activity increased significantly in the mixture group (the TMS + Al group). Regarding the fish that were analyzed 96 h after exposure, the one-way ANOVA revealed a significant decrease in the SOD (F (3.33) = 7.359; *p* = 0.0007) and GST (F (3.34) = 8.285; *p* = 0.0003) activities in the animals exposed to TMS and/or Al (the TMS, Al, and TMS + Al groups) when compared to those of the control group (Sal). The GPx activity was reduced significantly in the Al group (F (3.33) = 2.480; *p* = 0.0783) 96 h after exposure.

### 3.3. Principal Component Analysis (PCA)

**Figure 5:**
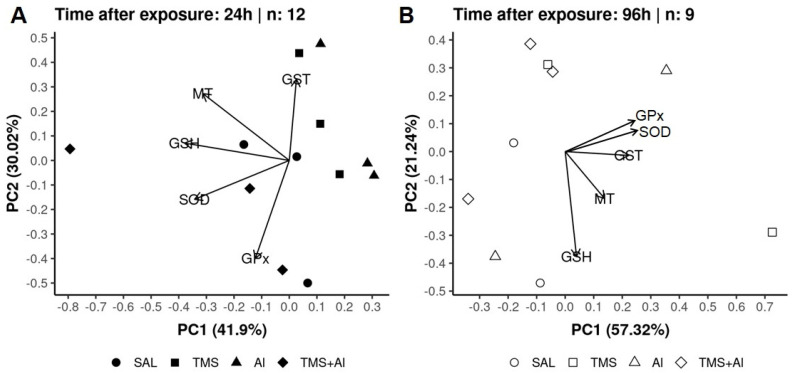
Liver principal component analysis of *D. rerio* exposed intraperitoneally to Sal (saline, 0.9%), TMS (thimerosal, 7.5 mg/kg), Al (aluminum hydroxide, 175.0 mg/kg), and TMS + Al (7.5 mg TMS/kg + 175.0 mg Al/kg) 24 h (**A**) and 96 h (**B**) after exposure.

In the PCA of the *D. rerio* livers using the biomarkers SOD, GST, GPx, GSH, and MT 24 h after exposure, axis 1 explained 41.9% and axis 2 explained 30.02% of the data variability. On the other hand, 96 h after exposure, axis 1 explained 57.32% and axis 2 explained 21.24% of the variability. Thus, the two axes explained 71.92% of the variability 24 h after exposure and 78.56% 96 h after exposure. From the graphic presentation (Figure 5A,B), it was possible to observe that the exposure groups (Sal, TMS, Al, and TMS + Al) after both exposure times did not differ, as observed in the univariate analysis.

**Figure 6:**
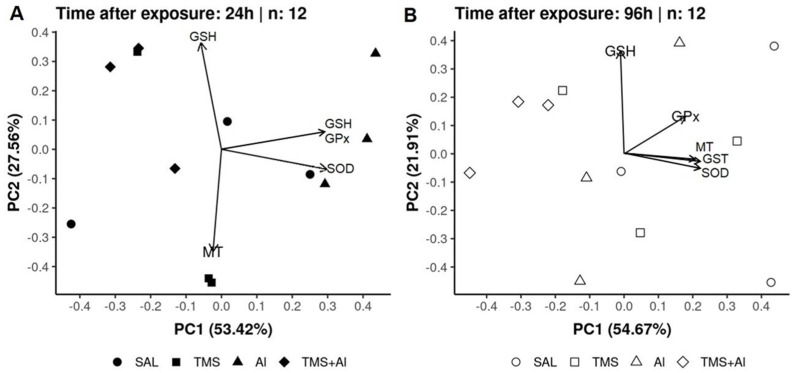
Kidney principal component analysis of *D. rerio* exposed intraperitoneally to Sal (saline, 0.9%), TMS (thimerosal, 7.5 mg/kg), Al (aluminum hydroxide, 175.0 mg/kg), and TMS + Al (7.5 mg TMS/kg + 175.0 mg Al/kg) 24 h (**A**) and 96 h (**B**) after exposure.

In the PCA of the *D. rerio* kidneys using the biomarkers SOD, GST, GPx, GSH, and MT 24 h after exposure, axis 1 explained 53.42% and axis 2 explained 27.56% of the data variability. On the other hand, 96 h after exposure, axis 1 explained 54.67% and axis 2 explained 21.91% of the variability. Thus, the two axes explained 80.98% of the variability 24 h after exposure and 76.58% 96 h after exposure. From the graphic presentation (Figure 6A,B), it was possible to observe that the group exposed to Al tends to separate from the other groups 24 h after exposure, as observed in the univariate analysis. On the other hand, this pattern does not occur 96 h after exposure.

**Figure 7:**
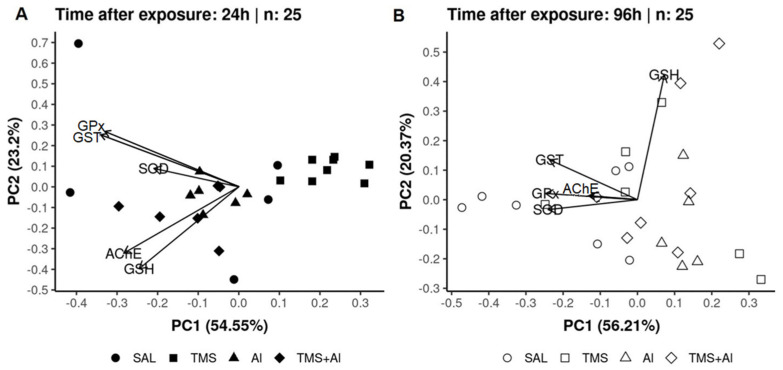
Brain principal component analysis of *D. rerio* brain exposed intraperitoneally to Sal (saline, 0.9%), TMS (thimerosal, 7.5 mg/kg), Al (aluminum hydroxide, 175.0 mg/kg), and TMS + Al (7.5 mg TMS/kg + 175.0 mg Al/kg) 24 h (**A**) and 96 h (**B**) after exposure.

In the PCA analysis of the *D. rerio* brains using the biomarkers SOD, GST, GPx, GSH, and AChE 24 h after exposure, axis 1 explained 54.55% and axis 2 explained 23.2% of the data variability. On the other hand, 96 h after exposure, axis 1 explained 56.21% and axis 2 explained 20.37% of the variability. Thus, the two axes explained 77.75% of the variability 24 h after exposure and 76.58% 96 h after exposure. From the graphic presentation (Figure 7A,B), it was possible to observe that the exposure only to the TMS (the TMS group) tends to separate from the other groups 24 h after exposure. Ninety-six hours after the exposure, the control (Sal group) tended to separate from the other groups, as observed in the univariate analysis.

### 3.4. Histopathological Analysis

**Figure 8:**
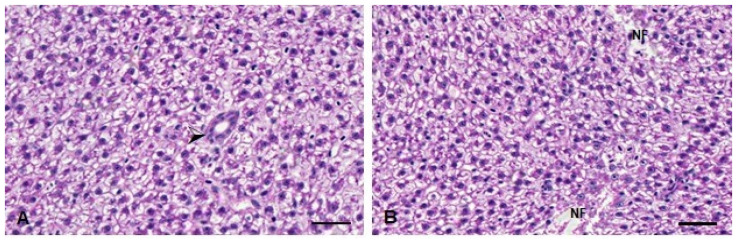
Liver histopathological analysis of *D. rerio* exposed intraperitoneally to (**A**) Sal (saline, 0.9%) and (**B**) TMS + Al (7.5 mg TMS/kg + 175.0 mg Al/kg) 96 h after exposure, counterstained with hematoxylin/eosin. (⮚) Hepatic parenchyma with bile duct. (NF) Necrotic focus. Scale bar = 50 µm.

In the histopathological analysis, no significant changes were observed in the *D. rerio* livers 24 h after exposure to the compounds. On the other hand, 96 h after exposure, the Kruskal–Wallis test revealed an effect of the metal exposure (H(3) = 15.16; *p* < 0.0001). The fish from the TMS + Al group have a statistically significant increase in the injury index 96 h after exposure (Table 1). The alteration observed in these animals was necrosis (Figure 8).

**Figure 9:**
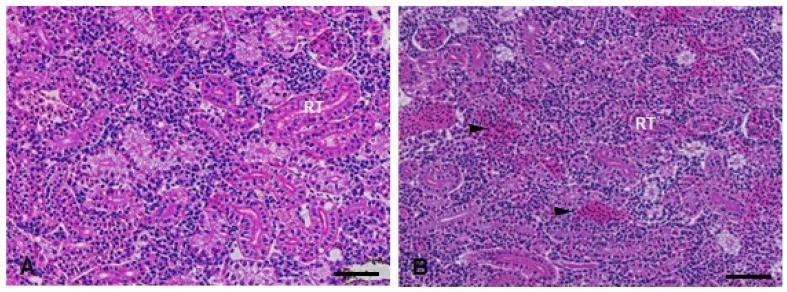
Kidney histopathological analysis of *D. rerio* exposed intraperitoneally to (**A**) Sal (saline, 0.9%) and (**B**) TMS + Al (7.5 mg TMS/kg + 175.0 mg Al/kg) 96 h after exposure, counterstained with hematoxylin/eosin. (RT) Parenchyma filled with renal tubules. (►) Hemorrhage in the parenchyma. Scale bar = 50 µm.

In the kidneys, the Kruskal–Wallis test showed a statistically significant increase in the injury index (H(3) = 10.95; *p* = 0.0120) in the TMS + Al group 24 h after exposure (Table 1). Ninety-six hours after exposure, the Kruskal–Wallis showed a statistically significant increase in the injury index (H(3) = 17.63; *p* = 0.0005) in the animals exposed to Al and TMS + Al (Table 1). Hemorrhage was the main alteration observed in both periods (Figure 9).

**Figure 10:**
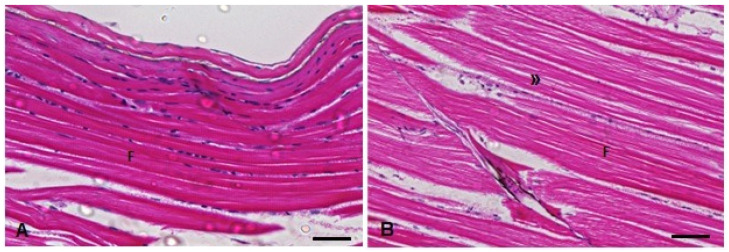
Muscle histopathological analysis of *D. rerio* exposed intraperitoneally to (**A**) Sal (saline, 0.9%) and (**B**) TMS + Al (7.5 mg TMS/kg + 175.0 mg Al/kg) 96 h after exposure, counterstained with hematoxylin/eosin. (**F**) Skeletal striated muscle fibers with visible striations. (**»**) Myofibril space in the muscle fibers. Scale bar = 50 µm.

The Kruskal–Wallis test showed a statistically significant increase in the muscle in-jury index (H(3) = 16.81; *p* = 0.0008) 96 h after exposure to TMS (the TMS and TMS + Al groups) (Table 1). The main histopathological alteration observed was the increase in the intramyofibril space (Figure 10).

**Figure 11:**
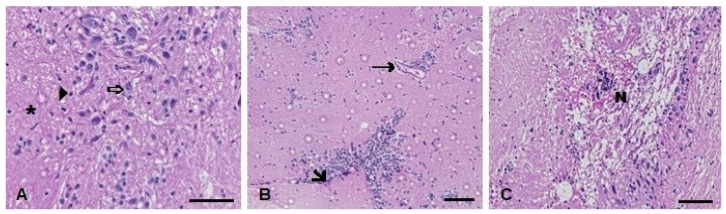
Muscle histopathological analysis of *D. rerio* exposed intraperitoneally to (**A**) Sal (saline, 0.9%) and TMS + Al (7.5 mg TMS/kg + 175.0 mg Al/kg) (**B**) 24 h and (**C**) 96 h after exposure, counterstained with hematoxylin/eosin. (⇨) Neurons. (►). (*) Neuropil (unmyelinated axons, dendrites, and glial cell processes). (**🡦**) Leukocyte infiltration. (→) Blood vessel. (N) Necrotic focus. Scale bar = 50 µm.

In the brain histopathological analysis, the Kruskal–Wallis’ test revealed a statistically significant increase in the injury index 24 h (H(3) = 8.362; *p* = 0.0391) and 96 h (H(3) = 12.08; *p* = 0.0071) after exposure to both metals (the TMS + Al group) (Table 1). The main alterations observed were necrosis and leukocyte infiltration (Figure 11).

### 3.5. Hg and Al Quantification

The one-way ANOVA revealed the effect of the metals on the whole-fish Hg levels at 24 h (F (3.16) = 12.04; *p* = 0.0002) and 96 h (F (3.13) = 18.25; *p* < 0.0001) after exposure. The fish exposed to TMS + Al had a statistically significant increase in their Hg levels 24 h after exposure; on the other hand, the fish exposed only to TMS (the TMS group) had a statistically significant increase in Hg levels 96 h after exposure when compared to those of the control group (Sal) (Table 2).

The one-way ANOVA revealed the effect of the metals on the whole-fish Al levels 24 h (F (3.16) = 16.93; *p* = < 0.0001) after exposure. The fish exposed to TMS + Al had a statistically significant increase in their Al levels 24 h after exposure. There was no significant difference in the Al levels 96 h after exposure (Table 2).

The body and head Hg and Al levels are shown in Table S3.

## 4. Discussion

There is a lack of studies on the toxicological effects of thimerosal and aluminum hydroxide, mainly in combination and in different tissues. These compounds are still used as components in pediatric vaccines [53,54], which has raised questions about their possible toxic mechanisms. It is important to emphasize that this is a primary and mechanistic study, and the dose of choice was much higher than the dose used in mandatory vaccines for children aged 0 to 10 years in Brazil. For example, FDA standards state that current vaccines in the US have <1 μg/kg of thimerosal in childhood vaccines, six times less than the dose applied in this study [55].

In this study, a decrease in MT levels was observed in the liver of *D. rerio* exposed to TMS + Al 96 h after exposure. Interestingly, studies have shown that exposure to other Hg chemical forms (mainly methylmercury) increases the hepatic MT mRNA expression [56,57] in fish. MT is a small protein with a high affinity for bivalent metal, playing a protective role in the body due to metal sequestration [58]. A decrease in GSH levels in the TMS + Al group was also observed in the *D. rerio* liver. According to Meister [59], GSH participates in the inactivation of free radicals, being one of the main cellular protective mechanisms. Thus, the decrease in GSH levels may lead to an increase in reactive oxygen species and, consequently, to oxidative stress [60,61]. Corroborating with our study, a decrease in hepatic GSH levels was also observed in rats exposed to thimerosal for 30 days, suggesting that this compound can cause damage to this organ [62]. The hepatic biochemical findings corroborate the hepatic histopathological analysis. The exposure to TMS + Al caused hepatic necrosis, a multi-step process that prevents cells from performing vital functions [63].

In the *D. rerio* kidney, significant changes were observed in the components of the antioxidant system, namely SOD, GPx, GST, and GSH. The SOD enzyme is responsible for the dismutation of superoxide, one of the main reactive oxygen species, into hydrogen and hydrogen peroxide [64]. GPx is a cytosolic enzyme that catalyzes the reduction of hydrogen peroxide to water and oxygen, as well as catalyzes the reduction of peroxide radicals to alcohols and oxygen [65]. GST is responsible for the detoxification of electrophiles through its conjugation with GSH [66]. The activity of these enzymes increased significantly in the Al group 24 h after exposure, indicating the possible adaptive response of the organism. The increase in SOD activity was also observed in rats exposed to aluminum hydroxide administered orally once a day for seven days [67]. Moreover, the GSH levels decreased significantly 24 h after exposure to TMS, corroborating the study by Ijaz et al. [68], who obtained the same result in rat kidneys after TMS intramuscular injections. We also observed a decrease in SOD and GPx activities and MT levels after 96 h of exposure. Our results are also validated by a histopathological analysis, in which the TMS + Al caused kidney hemorrhage.

In the muscle of *D. rerio* exposed to TMS, an increase in the intramyofibril space was observed, which can cause greater instability in the movement of the fish, increasing its vulnerability. Moreover, this alteration can culminate in cellular degeneration [69]. This finding raises concerns, as most vaccines are given intramuscularly. No papers in the literature were found to corroborate this result. Therefore, further studies should be carried out for this change to be better understood.

In our brain analysis, we observed the inhibition in oxidative-stress-related enzymes SOD and GST 96 h after exposure to TMS and Al, alone or in combination. The study of Ohtawa et al. [67] corroborates our findings, demonstrating a decrease in cerebral SOD in rats after exposure to Al for seven days. Moreover, the GPx activity decreased 96 h after exposure in the Al group. The fish from the TMS group also showed some alterations, such as a decrease in GSH levels and the AChE activity 24 h after exposure. Similar to our results, a study with *Drosophila melanogaster* found a time-dependent decrease in AChE activity after exposure to TMS [37]. According to Mota et al. [70], AChE is the enzyme responsible for the hydrolysis of the neurotransmitter acetylcholine in cholinergic synapses, in which acetylcholine acts to transmit the message from one neuron to another. The data suggested that a decrease in this enzyme can lead to damage in cholinergic transmission [71]. The histopathological analysis presented leukocytosis infiltration (24 h after exposure) and necrosis (96 h after exposure). The compounds tested in this study were associated with changes in the biochemistry and histopathology of the brain [72].

The accumulation of whole-fish Hg was observed at 24 h (in the TMS and TMS + Al groups) and 96 h (in the TMS group) after exposure. Unlike our study, Gonzalez et al. [73] did not observe a significant accumulation of Hg when evaluating the liver, skeletal muscle, and brain of zebrafish exposed to MeHg for 7, 21, and 63 days via food. On the other hand, Monteiro et al. [74] observed an accumulation of Hg in *Brycon amazonicus* exposed to mercury chloride. Al, in turn, accumulated in the entire fish 24 h after exposure (the TMS + Al group). No results were found in the literature that corroborate our findings. However, one study showed an accumulation of Al in the hair of children who had been immunized with hepatitis B, triple bacterial vaccine, and meningococcal vaccines [31]. Interestingly, in our study, Hg accumulated more in the head of the fish and Al in the body.

Summarizing our results, the PCA analysis shows the percentage variance between the two analysis times after exposure. This analysis complements the graphs previously presented and corroborates with the other results.

This study had some limitations, as it was the first using *D. rerio* (as our experimental model) and the metallic compounds (TMS and Al) together, as well as our dose of choice. A high dose was maintained since it did not alter the survival of the fish and is justified because it was a mechanistic study. Another limitation was the time to measure the metal dosage after 24 h of exposure since the dose used was high and the toxic metals were probably still bioavailable or trapped in inactive forms. Thus, we highlight the importance of further studies using different doses/concentrations.

## 5. Conclusions

In conclusion, TMS and Al caused biochemical and histopathological changes in the liver, kidney, muscle, and brain of *D. rerio*. All tissues showed alterations, but it seems the kidney and brain were more sensitive to exposure to both metals (the TMS + Al group). We emphasize the lack of information on the subject of this paper, which is a concern since TMS and Al are still used in the pediatric vaccines offered in developing countries. Therefore, research must continue to be developed to increase these populations’ safety against the possible toxic effects of these components, especially together and in the long term. Despite the risk of non-vaccination being greater than the risk of adverse effects, a better understanding of these reactions will allow for, in addition to better diagnoses, a better selection of vaccines, reducing the level of distrust in these populations and increasing their acceptance of immunization programs.

## Figures and Tables

**Table 1:** Liver, kidney, muscle, and brain histopathological analysis of *D. rerio* exposed intraperitoneally to Sal (saline, 0.9%), TMS (thimerosal, 7.5 mg/kg), Al (aluminum hydroxide, 175.0 mg/kg), and TMS + Al (7.5 mg TMS/kg + 175.0 mg Al/kg) 24 h and 96 h after exposure.

Groups	24 h	96 h
	Liver	
Sal	0.0 (0.0, 0.0)	0.0 (0.0, 0.0)
TMS	3.0 (0.0, 6.0)	0.0 (0.0, 6.0)
Al	0.0 (0.0, 6.0)	0.0 (0.0, 6.0)
TMS + Al	6.0 (0.0, 6.0)	6.0 (6.0, 6.0) *
	Kidney	
Sal	0.0 (0.0, 0.0)	0.0 (0.0, 0.0)
TMS	0.0 (0.0, 3.0)	0.0 (0.0, 1.0)
Al	4.0 (4.0, 4.0)	4.0 (0.0, 4.0) *
TMS + Al	7.0 (4.0, 8.0) *	4.0 (3.0, 4.0) *
	Muscle	
Sal	0.0 (0.0, 0.0)	0.0 (0.0, 0.0)
TMS	2.0 (0.0, 4.0)	2.0 (0.0, 5.0) *
Al	0.0 (0.0, 0.0)	0.0 (0.0, 0.0)
TMS + Al	4.0 (1.0, 7.0)	4.0 (0.0, 4.0) *
	Brain	
Sal	0.0 (0.0, 0.0)	0.0 (0.0, 0.0)
TMS	0.0 (0.0, 0.0)	0.0 (0.0, 8.0)
Al	0.0 (0.0, 0.0)	0.0 (0.0, 0.0)
TMS + Al	3.0 (0.0, 8.0) *	7.0 (0.0, 9.0) *

The injury index was analyzed by the Kruskal–Wallis test followed by Dunn’s post-hoc -test and is presented as median ± interquartile range (*n* = 8–10). * = statistically different from the control group (Sal).

**Table 2:** Hg and Al levels in *D. rerio* exposed intraperitoneally to Sal (saline, 0.9%), TMS (thimerosal, 7.5 mg/kg), Al (aluminum hydroxide, 175.0 mg/kg), and TMS + Al (7.5 mg TMS/kg + 175.0 mg Al/kg) 24 h and 96 h after exposure.

Groups	Whole Fish (µg Hg/g Fish)	Whole Fish (µg Al/g Fish)
24 h		
Sal	0.03 ± 0.02	0.29 ± 0.27
TMS	0.19 ± 0.15	0.12 ± 0.12
Al	0.03 ± 0.01	0.21 ± 0.06
TMS + Al	0.38 ± 0.15 *	32.38 ± 17.48 *
96 h		
Sal	0.04 ± 0.01	0.18 ± 0.17
TMS	0.26 ± 0.08 *	0.12 ± 0.14
Al	0.05 ± 0.01	6053.0 ± 10,476.0
TMS + Al	0.05 ± 0.04	9.32 ± 8.41

Results were analyzed by one-way ANOVA test followed by Dunnett’s post-hoc test and are presented as mean ± standard deviation (*n* = 3–5). * statistically different from the control group (Sal).

## Data Availability

The data presented in this study are available in article and supplementary materials.

## Supplementary Materials

The following supporting information can be downloaded at: https://www.mdpi.com/article/10.3390/metabo13090975/s1, Table S1: Aluminum and thimerosal doses injected intraperitoneally in *D. rerio* for mortality test. Table S2: Amount of aluminum and thimerosal present in mandatory vaccines for children from 0 to 10 years of age, established by the National Immunization Program in Brazil. Table S3: Mercury and aluminum levels in Danio rerio body and head of zebrafish exposed intraperitoneally to Sal (saline, 0.9%), TMS (thimerosal, 7.5 mg/kg), Al (aluminum hydroxide, 175.0 mg/kg) and TMS+Al (7.5 mg TMS/kg + 175.0 mg Al/kg) 24 h and 96 h after the exposure.
