# Supplementary material for: Toxicological Effects of Thimerosal and Aluminum in the Liver, Kidney, and Brain of Zebrafish (Danio rerio)
Source: Metabolites. 2023 Aug 27;13(9):975. doi: 10.3390/metabo13090975 (PMC10537066; doi:10.3390/metabo13090975)
Supplement: Supplementary file 1 [file metabolites-13-00975-s001.zip › metabolites-2510069-supplementary.pdf]

Supplementary Material

Table S1. Aluminum and thimerosal doses injected intraperitoneally in *D rerio* for mortality test.

	Aluminum	*Thimerosal*
Group 1	0 mg/kg	0 mg/kg
Group 2	0.28 mg/kg	-
Group 3	1.40 mg/kg	-
Group 4	7.00 mg/kg*	-
Group 5	35.00 mg/kg	-
Group 6	175.00 mg/kg	-
Group 7	-	0.012 mg/kg
Group 8	-	0.06 mg/kg
Group 9	-	0.30 mg/kg*
Group 10	-	1.50 mg/kg
Group 11	-	7.50 mg/kg

* Sum of known doses used in mandatory vaccines for children from 0 to 10 years of age ANVISA/Electronic Bulletin. (2013)

Table S2. Amount of aluminum and thimerosal present in mandatory vaccines for children from 0 to 10 years of age, established by the National Immunization Program in Brazil.

	Aluminum	*Thimerosal*	*Doses to be applied*	*Total of aluminum*	*Total of thimerosal*
Hepatitis B (0.5 mL)	0.025 mg	0.01% p/v	3 doses	0.075 mg	0.03% p/v
Triple bacterial	Up to 1.25 mg	Up to 0.05 mg	3 doses + 2 booster doses	Up to 6.25 mg	Up to 0.25 mg
Haemophilus influenzae type B	Does not contain	Uninformed*	3 doses + 1 booster dose	Does not contain	-
Influenza (0.5 mL)	Does not contain	2 ug	2 doses	Does not contain	4 ug
Conjugated pneumococcal	Uninformed*	Does not contain	2 ou 3 doses + 1 booster dose	-	Does not contain
Meningococcal B	0.5 mg	Does not contain	2 doses + 1 booster dose	1 mg	Does not contain
Hepatitis A	Uninformed*	Does not contain	2 doses	-	Does not contain
HPV	Uninformed*	Does not contain	2 doses	-	Does not contain
Triple acellular bacterial (adult type)	Uninformed*	Does not contain	1 dose	-	Does not contain

Source: ANVISA/Electronic Bulletin (2013)

* The exact amount of excipient in each dose is not informed in the package insert.

Table S3. Mercury and aluminum levels in *Danio rerio* body and head of zebrafish exposed intraperitoneally to Sal (saline, 0.9%), TMS (thimerosal, 7.5 mg/kg), Al (aluminum hydroxide, 175.0 mg/kg) and TMS+Al (7.5 mg TMS/kg + 175.0 mg Al/kg) 24h and 96h after the exposure.

Groups	Head (µg Hg/g fish)	Head (µg Al/g fish)	Body (µg Hg/g fish)	Body (µg Al/g fish)
		24 h		
Sal	0.12 ± 0.07	2.25 ± 2.00	0.13 ± 0.09	0.07 ± 0.09
TMS	1.46 ± 1.22	1.06 ± 1.50	0.34 ± 0.31	0.17 ± 0.24
Al	0.23 ± 0.20	2.33 ± 0.90	0.04 ± 0.06	1.48 ± 2.10
TMS + Al	1.02 ± 0.12	2.01 ± 2.20	1.36 ± 1.63	195.8 ± 180.4
		96h		
Sal	0.28 ± 0.0	0.64 ± 0.57	0.02 ± 0.03	0.51 ± 0.72
TMS	1.99 ± 0.40	0.58 ± 0.53	0.40 ± 0.07	0.66 ± 0.94
Al	0.25 ± 0.11	13.78 ± 17.33	N.D.	28,323.0 ± 40,055.0
TMS + Al	0.36 ± 0.28	1.80 ± 0.22	0.15 ± 0.10	79.47 ± 112.4

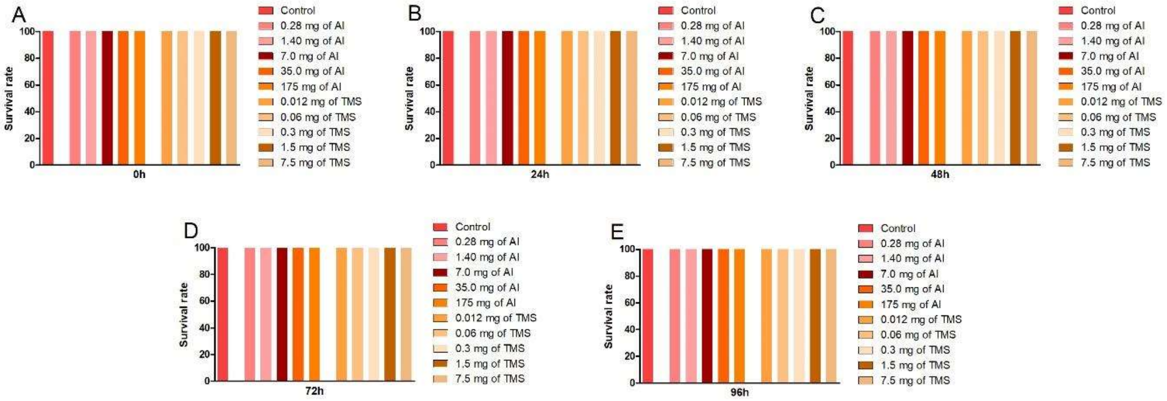

Figure S1. Survival rate of zebrafish followed until 96h after exposed to aluminum and thimerosal
